# The reduced growth due to elevated CO_2_ concentration hinders the sexual reproduction of mature Northern pipevine *(Aristolochia contorta* Bunge*)*

**DOI:** 10.3389/fpls.2024.1359783

**Published:** 2024-03-20

**Authors:** Si-Hyun Park, Jae Geun Kim

**Affiliations:** ^1^ Department of Biology Education, Seoul National University, Seoul, Republic of Korea; ^2^ Center for Education Research, Seoul National University, Seoul, Republic of Korea

**Keywords:** climate change, CO_2_ concentration, growth inhibition, phenology, reproduction, rhizome direction

## Abstract

The phenology has gained considerably more attention in recent times of climate change. The transition from vegetative to reproductive phases is a critical process in the life history of plants, closely tied to phenology. In an era of climate change, understanding how environmental factors affect this transition is of paramount importance. This study consisted of field surveys and a greenhouse experiment on the reproductive biology of Northern pipevine (*Aristolochia contorta* Bunge). During field surveys, we investigated the environmental factors and growth characteristics of mature *A. contorta*, with a focus on both its vegetative and reproductive phases. In its successful flowering during the reproductive phase, *A. contorta* grew under the conditions of 40% relative light intensity and 24% soil moisture content, and had a vertical rhizome. In the greenhouse experiments, we examined the impact of increased CO_2_ concentration on the growth and development of 10-year-old *A. contorta*, considering the effect of rhizome direction. Planted with a vertical rhizome direction, *A. contorta* exhibited sufficient growth for flowering under ambient CO_2_ concentrations. In contrast, when planted with a horizontal rhizome direction, it was noted to significantly impede successful growth and flowering under elevated CO_2_ concentrations. This hindered the process of flowering, highlighting the pivotal role of substantial vegetative growth in achieving successful flowering. Furthermore, we observed a higher number of underground buds and shoots under the conditions of elevated CO_2_ concentration and a horizontal rhizome direction instead of flowering. Elevated CO_2_ concentrations also exhibited diverse effects on mature *A. contorta*’s flower traits, resulting in smaller flower size, shorter longevity, and reduced stigma receptivity, and pollen viability. The study shed light on elevated CO_2_ concentrations can hinder growth, potentially obstructing sexual reproduction and diminishing genetic diversity.

## 1 Introduction

Climate change is a critical global issue with far-reaching consequences for the natural environment (Kumar et al., 2020). Climate change poses numerous challenges to plant species, influencing their biology, including their growth and reproductive strategies (Piao et al., 2019; Pareek et al., 2020). Phenology has garnered significant interest in recent years (Piao et al., 2019; Shen et al., 2022). Phenological shifts, which involve the timing of key events in plant life cycles such as flowering, fruiting, leafing out, and senescence, represent some of the most visible ecological effects of global climate change (CaraDonna et al., 2014). Among these phenological events, flowering naturally serves as a significant indicator of the transitions between vegetative and reproductive phases and is important in maintaining ecological balance (Schwartz, 2003). Climate change crucially modifies flowering time, which is a vital adaptation for ensuring successful reproduction in response to shifting environmental conditions (Cortés-Flores et al., 2017; König et al., 2017). There are various types of evidence indicating that phenological traits can be changed rapidly when there is strong natural selection which is influenced by environmental factors (Schwartz, 2003; Elzinga et al., 2007; Chuine, 2010). For example, elevated CO_2_ concentrations may offer certain benefits as they enhance photosynthesis, increase the number of flowers and fruits, and promote blooming (Chaudhry and Sidhu, 2022). However, other plants’ changes in CO_2_ concentration can disrupt the delicate balance of plant reproductive timing, potentially affecting reduced seed production (Boyle and Bronstein, 2012; Nam et al., 2020).

In response to the challenges posed by climate change, certain plant species have displayed adaptations in their flowering times, with certain species flowering earlier or later than usual to align with the changing climate (Inouye, 2020; Rafferty et al., 2020). These shifts in flowering patterns enable them to optimize their reproductive success in response to altered seasonal cues and environmental conditions (Rafferty et al., 2020; Rosbakh et al., 2021). Furthermore, some species exhibit modified flowering patterns, adjusting the timing of flowering to coincide with periods of increased pollinator availability (Inouye, 2020; Martins et al., 2021; Faust and Iler, 2022). Many other plants have evolved specialized underground structures that exhibit a remarkable ability to adjust their asexual reproductive structure in response to specific characteristics of the environment (Karlova et al., 2021; Huang et al., 2022). This adaptation is particularly advantageous when the ability to sprout and establish new growth from the rhizomes becomes a crucial strategy for plant resilience (Ziska et al., 2004; McPeek and Wang, 2007). Understanding how plants respond and adapt to environmental factors is vital for predicting their long-term survival, maintaining ecosystem stability, and contributing to overall biodiversity (Cleland et al., 2007; Sun and Frelich, 2011; Davies et al., 2013). Moreover, identifying the specific factors that drive the reproduction and investigating how selection by these factors impacts the most advantageous are crucial aspects of studying the evolution of an organism’s life history (Raza et al., 2020; Pesendorfer et al., 2021).

Northern pipevine *(Aristolochia contorta* Bunge*)* has garnered attention for research on climate change impacts. This perennial herbaceous vine plant is found in fragmented natural populations along forest edges and riverbanks in East Asia (Nam et al., 2020). It employs both sexual and asexual reproductive mechanisms (Lee, 2012). During sexual reproduction, this plant typically produces flowers after three or more years of growth (Park et al., 2019). The unique features of its flowers include a straight tubular perianth, enclosing fused styles, stigma, and anthers within a chamber known as the utricle, which forms a gynostemium (Park and Kim, 2023). During asexual reproduction, the plant stores reserves in the root or rhizome and undergoes seasonal changes, shedding its aboveground parts in winter and regrowing new stems from underground buds annually (Lee, 2012). Additionally, the vulnerable butterfly, *Sericinus montela*, whose larvae feed solely on *A. contorta*, underscores the importance of conservation studies (Park et al., 2023).

Previous studies of *A. contorta* have mainly focused on its functional aspects, such as plant’s secondary metabolites and its optimal habitat conditions (Hashimoto et al., 1999; Cheung et al., 2006; Heinrich et al., 2009; Nakonechnaya et al., 2013; Park et al., 2019). Studies have shown that the genetic variation indices of *A. contorta* are low, similar to those of other rare plants (Nakonechnaya et al., 2012; Nam et al., 2020). Elevated CO_2_ concentrations inhibit the growth of 1- or 2-year-old *A. contorta*, decrease photosynthesis, and increase plant resistance, negatively impacting specialist herbivores (Park et al., 2021). The effect of climate change on the interaction between *A. contorta* and its specialist and generalist herbivores vary depending on the ontogenetic stage of the plant (Park et al., 2022). However, previous studies have focused only on *A. contorta* growth during the vegetative phase, and no research has been conducted on its reproductive phase. Furthermore, there is a lack of research on the reproductive biology of this plant under climate change.

To address this gap, we investigated the growth and reproductive characteristics of mature *A. contorta* under different CO_2_ concentrations while also examining trade-off patterns in its reproductive strategies. The field survey provided valuable insights into the conditions for flowering through a comparison of the vegetative and reproductive phases, and the greenhouse experiment was guided by the findings from the field survey to identify the factors hindering or triggering flowering. We hypothesized that (1) the elevated CO_2_ concentration will impede the growth of mature *A. contorta*, which in turn will hinder flowering and lead to a transition from sexual reproduction to asexual reproduction, and (2) the impact of elevated CO_2_ concentration will be more pronounced when the rhizome direction of mature *A. contorta* is horizontal. This research underscores the novel contributions of our study within the broader context of the impacts of climate change on plant species, with a particular focus on *A. contorta*. Delving into the relatively unexplored domain of its reproductive biology under varying CO_2_ conditions, our investigation provides critical insights into the adaptive responses of mature *A. contorta* to climate change and its reproductive behavior. Such knowledge is pivotal in guiding conservation efforts, aiding in mitigating the impact of climate change on *A. contorta* populations, preserving their genetic diversity, and ensuring their long-term survival.

## 2 Materials and methods

### 2.1 Vegetative and reproductive phases comparison

#### 2.1.1 Environmental factor analysis

The research was conducted in Anyang, Gyeonggi Province, South Korea (37°24’2.58” N, 126°58’18.3” E) during the flowering period from May to August in, 2023, documented a mean temperature of 24.82 ± 0.29°C and a mean precipitation of 16.07 ± 3.23 mm. For the region, the 30-year annual mean temperature from, 1993 to, 2022 was 12.60 ± 0.10°C, and the annual mean precipitation was, 1337.01 ± 52.86 mm (Korea Meteorological Administration, 2023). The site was situated in a riparian area within the native distribution range of *A. contorta*. Relative light intensity (RLI) was measured by comparing the light intensity (µmol m^−2^ s^−1^) recorded at the top of *A. contorta* with that measured at the same height in an open space at the same time (Lee and Cho, 2000). We measured soil properties, including water content, pH, EC, PO_4_–P, NH_4_–N, NO_3_–N, Ca^2+^, K^+^, Na^+^, and Mg^2+^. Analyses of soil properties were performed by obtaining soil samples from a depth of 15 - 25 cm near the rhizomes of each individual *A. contorta*. To preserve moisture, the samples were sealed in plastic bags and transported to the laboratory. The soil samples were then sieved through a 2 mm mesh, and a mixture of deionized water was added at a ratio of soil 1: water 5. The resulting solution was filtered using Whatman filter paper No. 42 (Sigma-Aldrich, St. Louis, MO, USA) and used for analyses of soil environmental characteristics. The pH level was determined using a pH meter (model AP 63; Fisher Scientific, Pittsburgh, PA, USA), and the electrical conductivity (EC) was measured using a conductivity meter (Corning Checkmate model 311; Corning Incorporated, Tewksbury, MA, USA). For soil nutrients, we determined the contents of PO_4_–P, NH_4_–N and NO_3_–N using the methods of hydrazine (Kamphake et al., 1967), indo-phenol (Murphy and Riley, 1962), and ascorbic acid reduction (Solorzano, 1969), respectively. Exchangeable cations (Ca^2+^, K^+^, Na^+^ and Mg^2+^) were measured using an atomic absorption spectrometer (model AA240FS; Varian Medical Systems, Palo Alto, CA, USA) after extraction with 1 M ammonium acetate solution. Additionally, soil water content was determined by drying the fresh soil samples at 105°C for more than 48 hours (Kim et al., 2004), while soil organic matter contents were analyzed using loss on ignition at 450°C (John, 2004).

#### 2.1.2 Growth trait analysis

During the transition from vegetative phase to reproductive phase (when the flower buds began to appear), 20 individuals (older than 4 years; approximately 2 m stem length, the length of the onset of flowering according to observations in field survey) were taken into account for both the flowering group, consisting of 10 individuals spaced more than 10 meters apart, and the non-flowering group of 10 individuals. The measured growth factors were stem thickness at ground level, internode length, number of branches, number of leaves, single leaf area, total leaf area, rhizome thickness, rhizome length, direction of rhizome growth, chlorophyll content, fresh and dry weights (stems, leaves, rhizomes with roots, and flowers), and C/N ratios of each part (stem, leaves, and rhizome). Stem and rhizome thickness were measured with a vernier calipers (Mitutoyo, Kanagawa, Japan; resolution, 0.01 mm). The first internode length above the ground and number of leaves were recorded for each individual. To calculate the total leaf area, we first determined the leaf area for each individual by measuring the average area of ten leaves using ImageJ (Schneider et al., 2012). Subsequently, the average leaf area was applied to the total number of leaves. For assessing rhizome direction, we excavated the soil to a depth of approximately 20 cm. We utilized an angle gauge to measure the angle between the rhizomes and the horizontal plane, providing a clear indication of their orientation. In order to gauge the chlorophyll content of leaves, we employed a chlorophyll meter (SPAD-502, Konica Minolta, Tokyo; Rodriguez and Miller, 2000). Moreover, the dry weights of stems, leaves, and rhizomes with roots were measured, and belowground/aboveground ratio was calculated by dividing the dry weight of the rhizome with roots by the dry weight of the aboveground shoot components of a plant. In order to investigate the distribution of carbon and nitrogen resources in various plant parts, we performed stoichiometric analyses of the rhizomes, stems, leaves, flowers, and fruits. The plant parts were dried in a dry oven at 60°C and then ground using a ball mill (Pulverisette 23; Fritsch, Germany) to ensure uniform mixtures for the analysis. C/N ratio of each part was measured using an elemental analyzer (Flash EA, 1112, Thermo Electron, USA) at the National Instrumentation Center for Environmental Management (NICEM) at Seoul National University.

### 2.2 Effects of elevated CO_2_ on growth and reproduction in different rhizome directions

#### 2.2.1 Experimental design

To investigate how individuals with different inclinations respond to elevated CO_2_ concentrations, we conducted greenhouse experiments to elucidate the changes in the growth and reproduction of *A. contorta* under different CO_2_ concentrations. For this purpose, we purchased 10-year-old *A. contorta* rhizomes in March 23, 2023.

We observed that growth and reproductive characteristics differed based on whether the rhizomes were oriented vertically or horizontally in the field surveys. To replicate the natural conditions closely, we planted a total of 40 rhizomes of these plants individually, both horizontally and vertically, in 5 liters of soil each (Superlative soil, Gumok, Pohang-si, Republic of Korea; Park et al., 2019). The greenhouse, located at Seoul National University, Seoul, Republic of Korea, provided a relative light intensity of 37.9% (Park et al., 2019). We installed hexagonal Open Top Chambers (OTC; Park et al., 2021) in the greenhouse to manipulate the carbon dioxide (CO_2_) concentration, simulating two scenarios (Thomson et al., 2011): 1) Representative Concentration Pathways 4.5 (RCP 4.5) climate change scenario with a CO_2_ concentration of 540 ppm, and 2) current ambient conditions with a CO_2_ concentration of 400 ppm. Each OTC had its own CO_2_ control system to regulate elevated CO_2_ concentration. The control system consisted of a sensor-transmitter coupled with a CO_2_ controller (SH-MVG260, Soha-tech, Korea), capable of maintaining CO_2_ concentrations within the range of 0 – 2000 ppm. Additionally, a solenoid valve and individual CO_2_ gas tanks (40 L, 99.999% purity) were used in the setup (Park et al., 2021). Therefore, we conducted a greenhouse experiment using a total of 40 plants, with 10 plants for each specific condition. The experiment involved two CO_2_ concentrations (ambient 400 ppm and elevated 540 ppm), and two rhizome directions planting (horizontal rhizome planting, H; and vertical rhizome planting, V), resulting in four experimental treatments (400ppmCO_2_/H, 400ppmCO_2_/V, 540ppmCO_2_/V, 540ppmCO_2_/H) arranged in a factorial design. Temperature and relative humidity sensors (HOBO Pro v2, Onset, Bourne, MA, USA) were installed in each chamber, and these variables were ensured to remain consistent across all chambers throughout the experimental period. The mean air temperature and humidity recorded in the OTCs during the experimental period were as follows: 24.3°C and 69.5% in ambient CO_2_, 24.1°C and 68.4% in elevated CO_2_.

Pots were set on 50 mm thickness plates to minimize external wind effects from the bottom of the chamber induced by the ventilation system. To measure the amount of nutrients absorbed by each plant, bottom watering was used. A rope wick was inserted into the base of the pots to facilitate water absorption from the tray below the pot. During harvesting, excess water at the bottom was directed into the soil to reduce nutrient loss. To compare the nutrient absorption efficiency based on the treatment conditions, we evaluated the differences in soil nutrient levels between before and after the experiment, including NO_3_–N, NH_4_–N, PO_4_–P, as well as cations (K^+^, Na^+^, Ca^2+^, and Mg^2+^), along with the C/N ratio. The calculation of nutrient uptake by plants and nutrient losses (efforts were made to minimize nutrient loss during irrigation, and while some leaching was expected, the uniform water supply likely resulted in consistent nutrient loss) involved subtracting the nutrient content in the soil after the experiment from that before the experiment.

#### 2.2.2 Analysis of growth and reproductive traits

To assess the growth and reproductive (sexual and asexual) traits of *A. contorta* under two CO_2_ concentrations and two rhizome direction plantings, we harvested all plants in the greenhouse at the initial signs of aboveground senescence. We separated the collected plants into stems, leaves, and flowers and measured the growth traits of stem length, internode length, stem thickness, number of branches, total branch length, number of leaves, single leaf area, total leaf area, chlorophyll content, changes in fresh and dry weight of each component (stem, leaf, rhizome and roots), and C/N ratios of each part (stem, leaves, rhizome and root). We assessed variations in reproductive traits related to sexual reproduction, including the first flowering day (FFD), number of flowers, flowering duration, flower longevity, perianth size, diameter of the utricle, pollen grain size, stigmatic receptivity, pollen viability, and C/N ratio of the perianth and ovary (the breeding process of *A. contorta* heavily relied on pollinators. Only one fruit was produced and we opted to measure the ovary instead). Flowering duration refers to the period from the FFD until the last flower wilts or fades, while flower longevity denotes the duration from the budding of a single flower until it withers. The sizes of the perianth, utricle, fruit, and seed were measured using a digital vernier calipers. Ten flowers were assessed under each condition, with ten pollen grain diameters measured from each individual flower. The sizes of pollen grains were measured using an optical microscope (DE/Axio Inager A1 microscope, Carl Zeiss, Germany) and ImageJ. For stigmatic receptivity assessment, we collected ten samples from each flowering individual on the first day of flower opening. Subsequently, we conducted separate applications of a 3% hydrogen peroxide (H_2_O_2_) solution to the stigmas of both the female and male phases (Dafni and Maués, 1998; Serrano and Olmedilla, 2012). The stigmas were observed for a duration of 3 minutes under a stereoscopic microscope (GB-742, Global4U, Republic of Korea), and the presence of bubbles served as a reliable indicator to assess their receptivity. Stigmas that displayed a substantial number of bubbles were classified as highly reactive to the compound, while those with minimal or no bubble formation were categorized as having low reactivity. It was assessed using the approach adapted from Dafni and Maués (1998), involving the assignment of scores based on the number of bubbles. These scores included no reaction (0), a weak positive reaction (1), a strong positive reaction (2), and a very strong positive reaction (3). Pollen viability was assessed using the 1% neutral red staining method (Georgieva and Kruleva, 1993). Three samples were collected from each flowering individual and prepared. Viability was calculated by counting stained (viable or semi viable) and unstained (non-viable) grains from ten samples from each flowering individual on the third day (after 48 h of opening, male phase) of flower opening. The number of underground buds and shoots were recorded as reproductive organs related to asexual reproduction. To explore the variations in the distribution of carbon and nitrogen resources under the two different CO_2_ concentrations, we conducted stoichiometric analyses on the rhizomes, stems, leaves, flowers, and fruits. The experimental methodology was consistent with the previously described procedure.

### 2.3 Statistical analyses

We employed an analysis of variance (ANOVA) after the homogeneity of variance test and post-hoc tests (Duncan’s test) to determine the significance of the observed variations in growth and reproductive parameters between the control and treatment groups. Canonical correlation analysis (CCA) was employed to confirm the relationships between environmental factors and growth traits (including the number of flowers) during the field survey, as well as between growth and reproductive traits in the greenhouse experiment. This analysis was performed using PC-ORD for Windows version 5 (B. McCune and MJ Mefford, MjM Software, Gleneden Beach, OR, USA). To comprehend the impact and interaction between rhizome direction and CO_2_ concentration, we conducted a multivariate analysis of variance (MANOVA). We utilized SPSS software version 23.0 (SPSS, Inc., Chicago, IL, USA) for statistical analysis, with the significance level set at *p* < 0.05.

## 3 Results

### 3.1 Vegetative and reproductive phases comparison

#### 3.1.1 Environmental factor analysis

**Figure 1:**
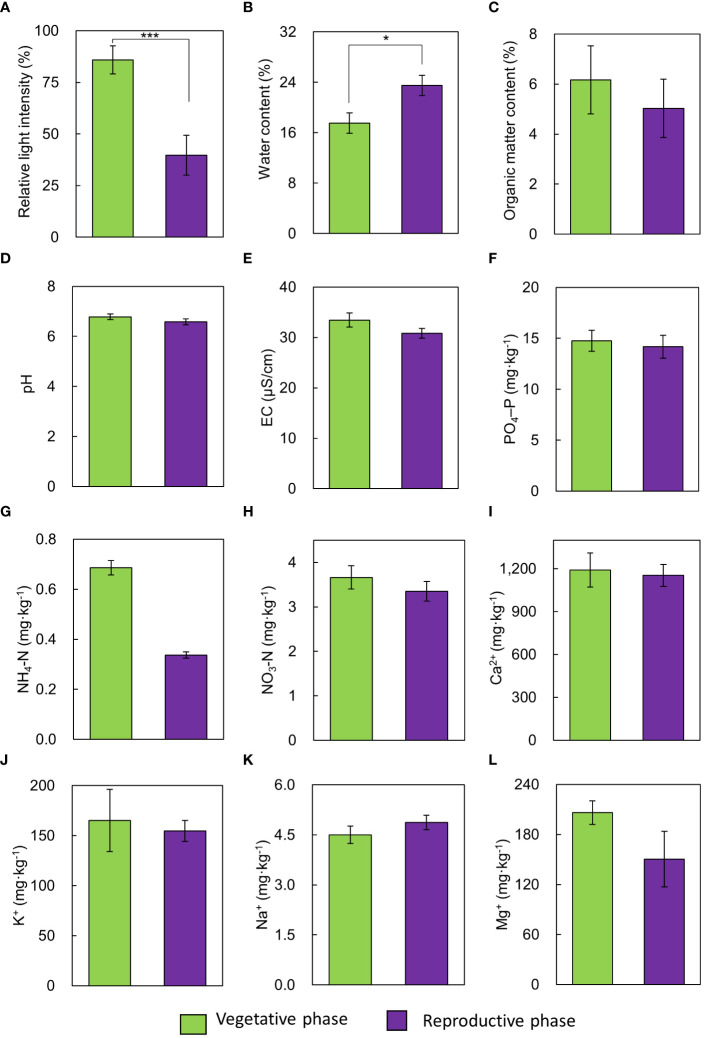
Comparison of environmental variables between vegetative and reproductive phases. **(A)** relative light intensity, **(B)** soil water content, **(C)** soil organic matter content, **(D)** pH, **(E)** EC, **(F)** PO_4_–P, **(G)** NH_4_–N, **(H)** NO_3_–N, **(I)** Ca^2+^, **(J)** K^+^, **(K)** Na^+^, **(L)** Mg^2+^. Bars indicate standard errors. *p < 0.05; ***p < 0.001.

Among the environmental factors, relative light intensity and soil water content showed statistically significant differences between the vegetative and reproductive phases (**Figure 1**), and there were no significant differences in other environmental factors. The vegetative phase had a higher RLI (85.91%) than the reproductive phase (39.74%, **Figure 1A**).

#### 3.1.2 Growth traits analysis

**Figure 2:**
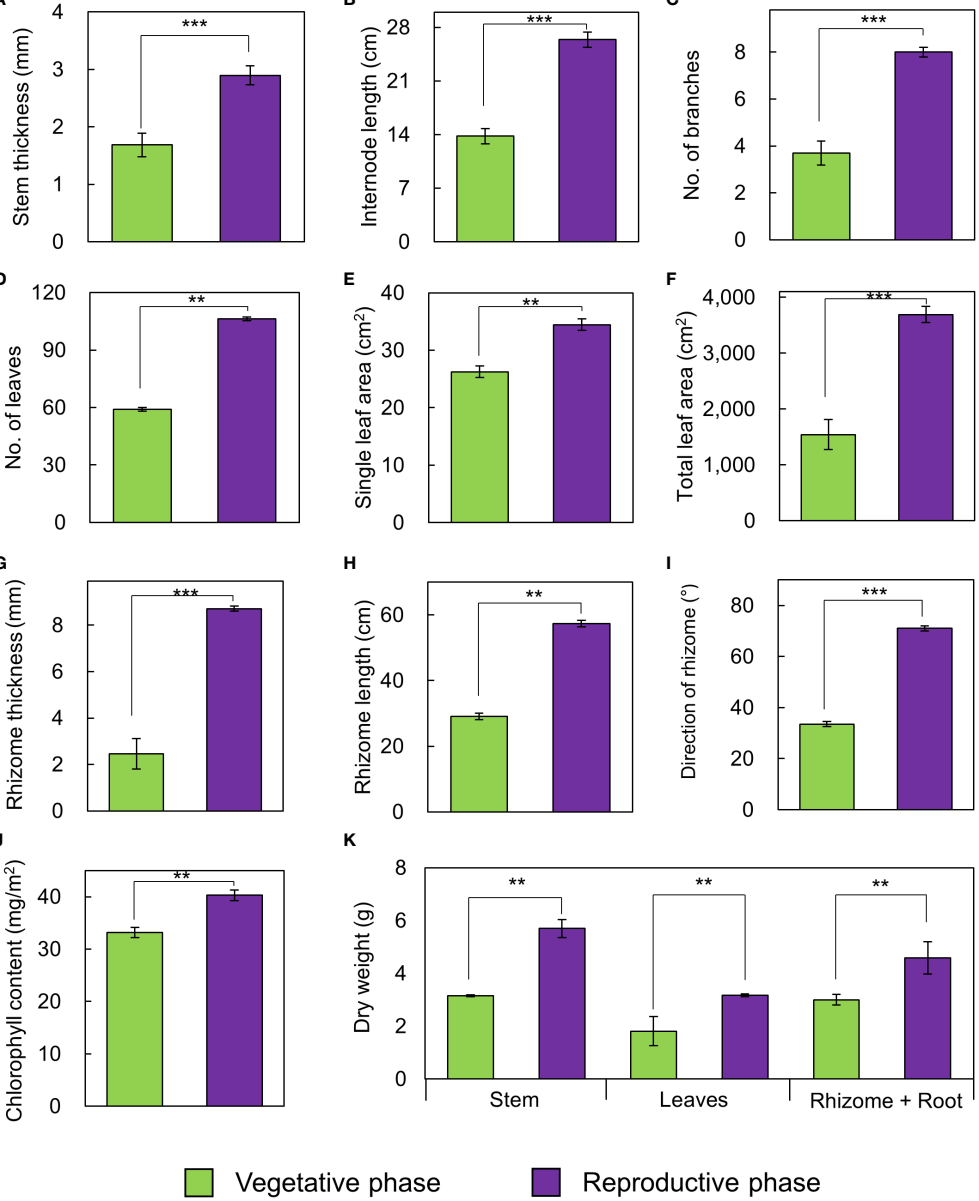
Growth traits of *A*. *contorta* in vegetative and reproductive phases. **(A)** stem thickness, **(B)** length of the first internode, **(C)** number of branches, **(D)** number of leaves, **(E)** single leaf area, **(F)** total leaf area, **(G)** rhizome thickness, **(H)** rhizome length, **(I)** direction of rhizome, **(J)** chlorophyll content, **(K)** dry weight of stem, leaves, rhizome and root. Bars indicate standard errors. **p < 0.01; ***p < 0.001.

**Figure 3:**
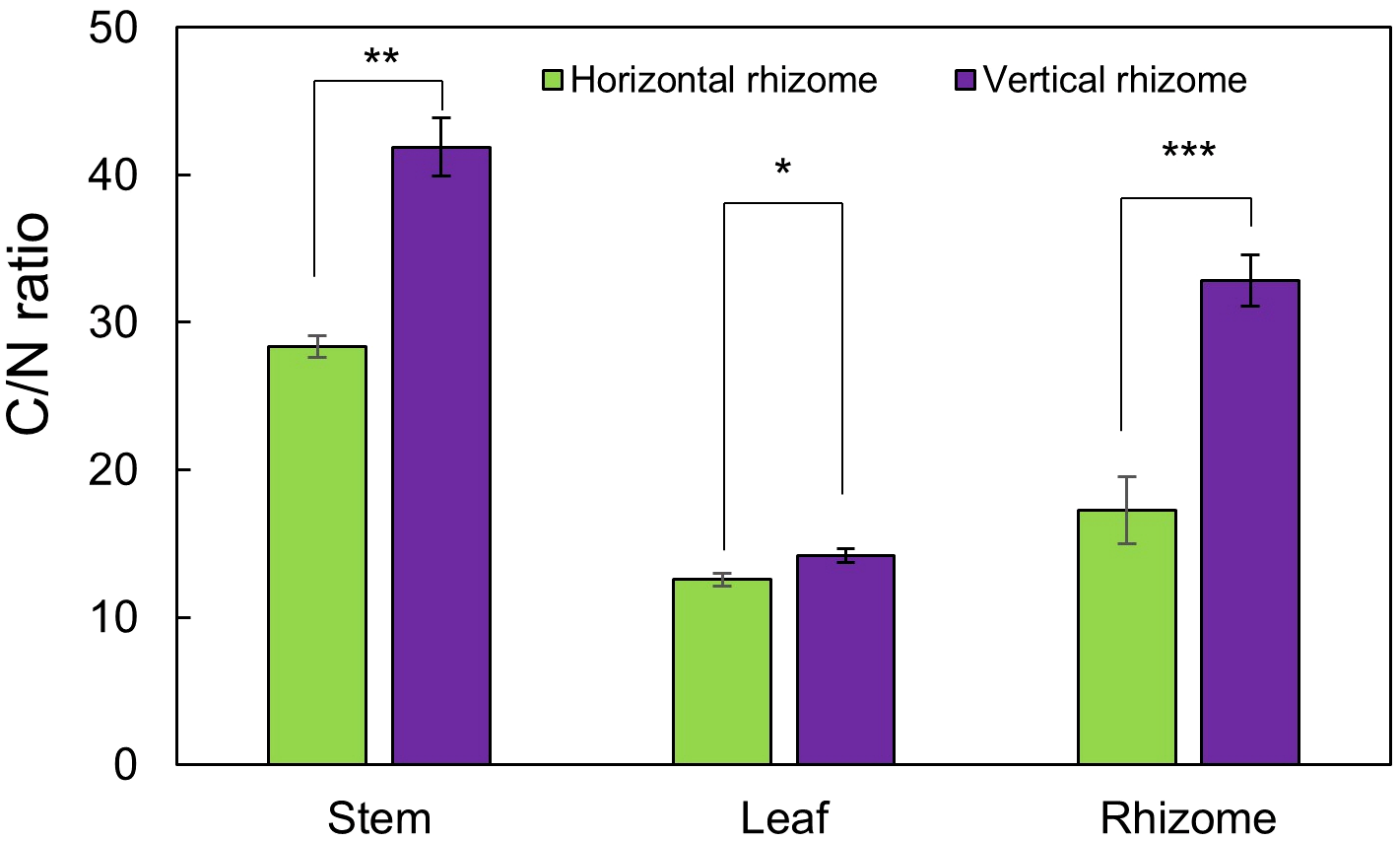
C/N ratio of each part in the vegetative phase and reproductive phase. Bars indicate standard errors. *p < 0.05; **p < 0.01; ***p < 0.001.

**Figure 4:**
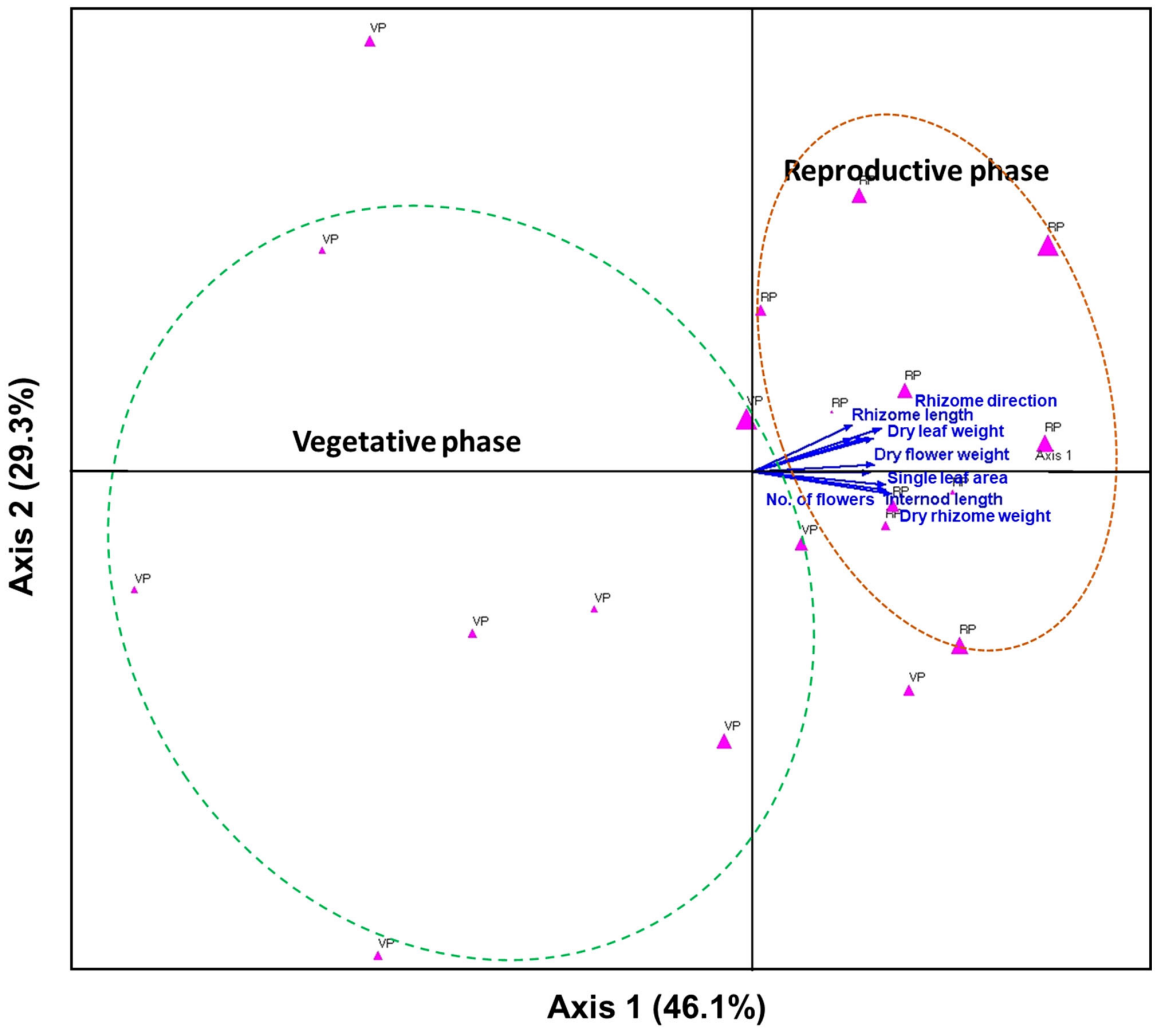
Canonical correlation analysis (CCA) plots to determine the relationships among the environmental factors and growth traits. The percentage (%) of each axis represents the explained variance. Dotted curves indicate groups of individuals in the vegetative and reproductive phases, which are represented by pink triangles. The arrows are strongly correlated with the axis.

The growth traits of *A. contorta* during the vegetative and reproductive phases were significantly different (**Figure 2**). Stems were thicker in the reproductive phase (2.90 ± 0.21 mm) than in the vegetative phase (1.69 ± 0.17 mm). Internode length was longer in the reproductive phase (26.4 ± 1.2 cm) than in the vegetative phase (13.8 ± 1.2 cm). Branch and leaf numbers per quadrat were more in the reproductive phase (8.0 ± 0.5 branches, 106.4 ± 9.1 leaves) than in the vegetative phase (3.7 ± 0.7 branches, 59.0 ± 13.8 leaves). Single leaf area (34.46 ± 1.35 cm^2^) and total leaf area (3690.80 ± 267.46 cm^2^) in reproductive phase were greater than those in vegetative phase (single leaf area, 26.25 ± 1.67 cm^2^; total leaf area, 1542.42 ± 460.30 cm^2^). There were broad and horizontal leaves in reproductive phase and small leaves pasted vertically on shorter internodes in vegetative phase. Rhizome thickness (8.71 ± 0.66 mm) and length (57.4 ± 5.9 cm) were greater at reproductive phase than (rhizome thickness, 2.46 ± 0.11 mm; root length, 29.1 ± 3.4 cm). Direction of root growth in vegetative phase was 33.5 ± 5.9°, and those in reproductive phase was 71.0 ± 5.6°. Chlorophyll contents in vegetative phase (33.16 ± 0.91 mg/m^2^) and reproductive phase (40.27 ± 1.63 mg/m^2^) were different. Dry weight of each part in reproductive phase (stem, 5.70 ± 0.33 g; leaves, 3.17 ± 0.11 g; rhizome and root, 4.59 ± 0.29 g) were significantly larger than those in vegetative phase (stem, 3.15 ± 0.27 g; leaves, 1.81 ± 0.11 g; rhizome and root, 2.99 ± 0.28 g; **Figure 2**). The belowground/aboveground ratio showed a ratio of 0.56 in vegetative phase and 0.51 in reproductive phase (*p*=0.070). C/N ratios were significantly different each part of the vegetative and reproductive phases. Each part in vegetative phase were: a stem, 28.34 ± 0.74; leaves, 12.55 ± 0.46; a rhizome, 17.23 ± 2.27; and those at reproductive phase were: a stem, 41.89 ± 1.97; leaves, 14.18 ± 0.45; a rhizome, 32.84 ± 1.73 (**Figure 3**). The environmental variables were correlated with internode length, single leaf area, rhizome length, direction of rhizome, dry leaf weight, dry rhizome weight, dry flower weight, and number of flowers (**Figure 4**). Axes 1 and 2 accounted for 46.1% and 29.3% of the explained variance, respectively.

### 3.2 Effects of elevated CO_2_ on growth and reproduction in different rhizome directions

#### 3.2.1 Nutrient uptake of *A. contorta* and soil environmental conditions

**Figure 5:**
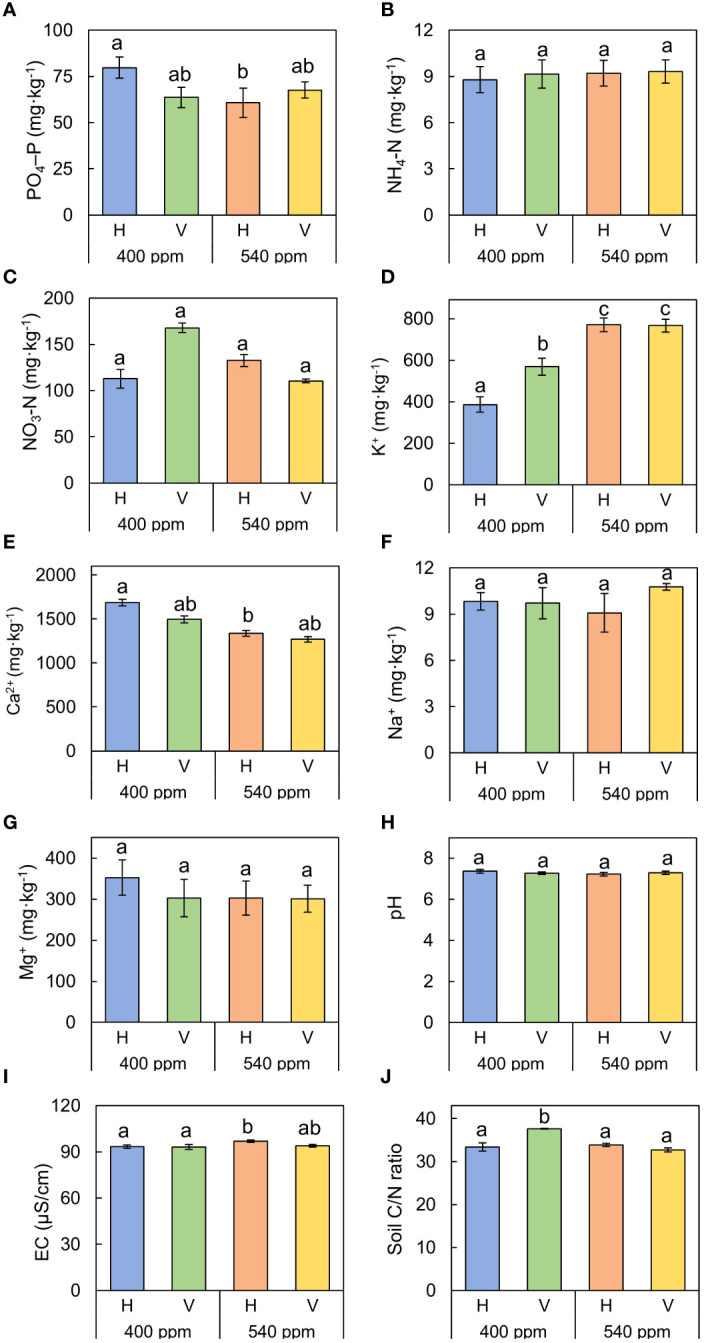
Sum of nutrient uptake by *A*. *contorta* and nutrient losses under two CO_2_ concentrations (400 ppm, 540 ppm), and two rhizome directions (horizontal rhizome planting, H; and vertical rhizome planting, V). **(A)** PO_4_–P, **(B)** NH_4_–N, **(C)** NO_3_–N, **(D)** Ca^2+^, **(E)** K^+^, **(F)** Na^+^, **(G)** Mg^2+^, **(H)** pH, **(I)** EC, **(J)** soil C/N ratio. Letters on the graph indicate significant differences at the 5% level, based on Duncan’s test. Bars indicate standard errors.

In the greenhouse experiment, there were no significant differences in nutrient uptake for any nutrient except for potassium (**Figure 5**). Absorbed potassium was significantly largest in the 540ppmCO_2_/H (770.85 ± 33.17 mg·kg^-1^), followed by 540ppmCO_2_/V (766.13 ± 30.13 mg·kg^-1^), 400ppmCO_2_/V (569.14 ± 40.99 mg·kg^-1^), and 400ppmCO_2_/H (386.17 ± 36.60 mg·kg^-1^).

In the case of soil C/N ratio, the significantly highest value was observed in 400ppmCO_2_/V (37.66 ± 0.09), followed by 540ppmCO_2_/H (33.84 ± 0.37), 400ppmCO_2_/H (33.40 ± 0.99), and 540ppmCO_2_/H (32.73 ± 0.47).

#### 3.2.2 Elevated CO_2_ and rhizome direction effects on growth and reproductive traits

**Figure 6:**
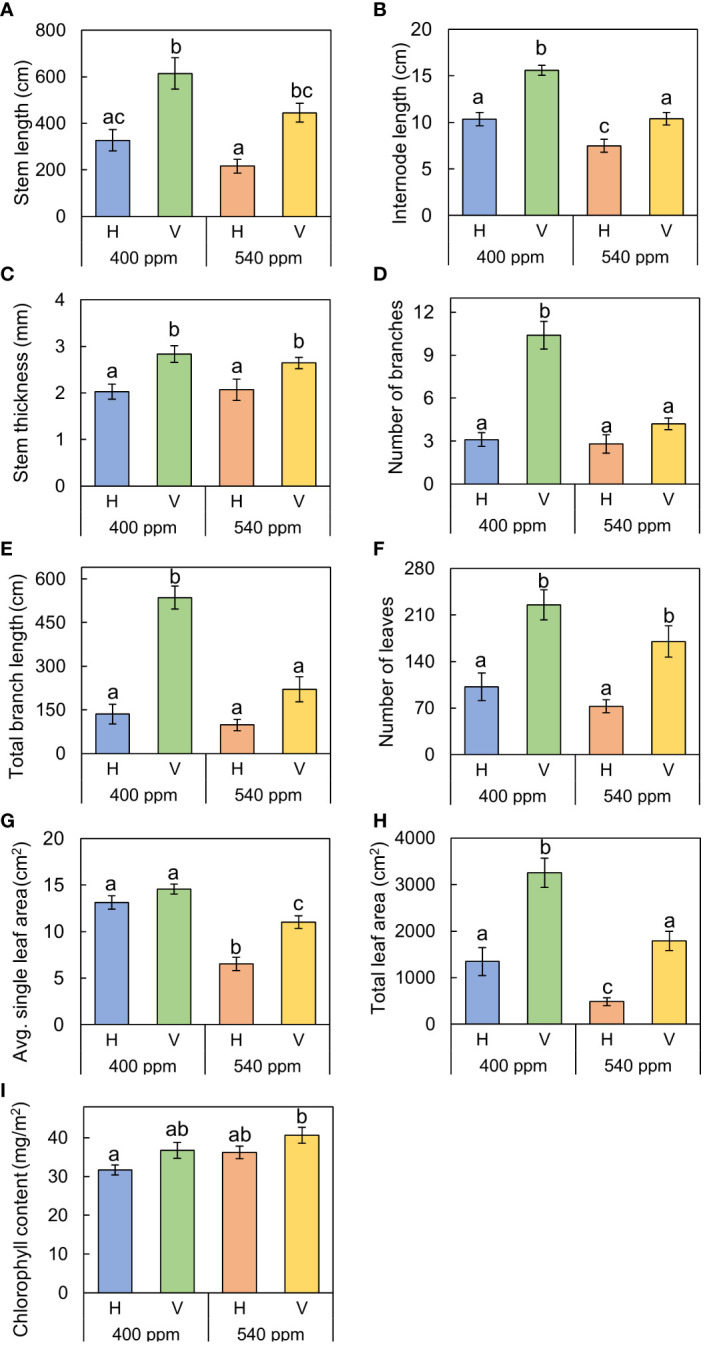
The growth traits of *A*. *contorta* under two CO_2_ concentrations (400 ppm, 540 ppm), and two rhizome directions (horizontal rhizome planting, H; and vertical rhizome planting, V). **(A)** stem length, **(B)** length of the first internode, **(C)** stem thickness, **(D)** number of branches, **(E)** total branch length, **(F)** number of leaves, **(G)** single leaf area, **(H)** total leaf area, **(I)** chlorophyll content. Letters on the graph indicate significant differences at the 5% level, based on Duncan’s test. Bars indicate standard errors.

**Figure 7:**
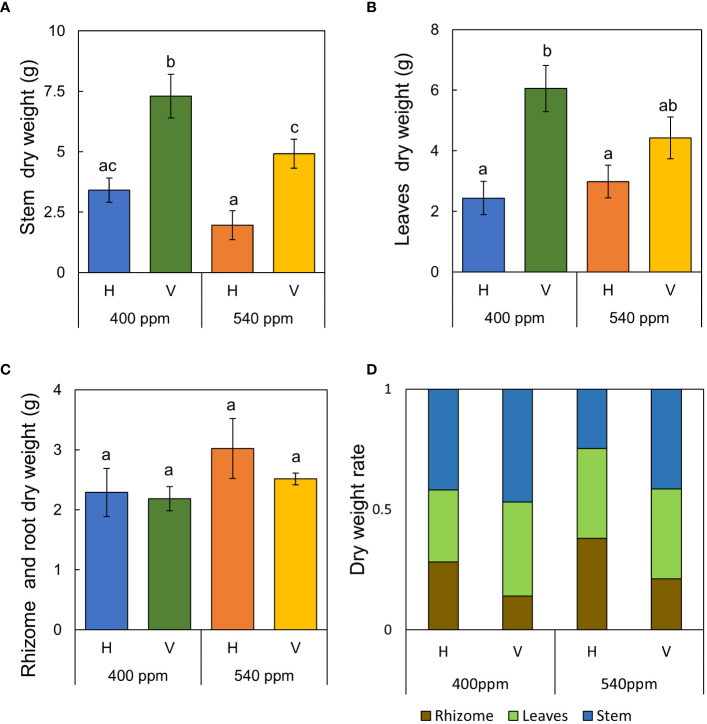
Dry weight of each part of *A*. *contorta* and allocation of dry weight under two CO_2_ concentrations (400 ppm, 540 ppm), and two rhizome directions (horizontal rhizome planting, H; and vertical rhizome planting, V). **(A)** stem weight, **(B)** leaves weight, **(C)** rhizome and root weight, **(D)** allocation of dry weight. Letters on the graph indicate significant differences at the 5% level, based on Duncan’s test. Bars indicate standard errors.

**Figure 8:**
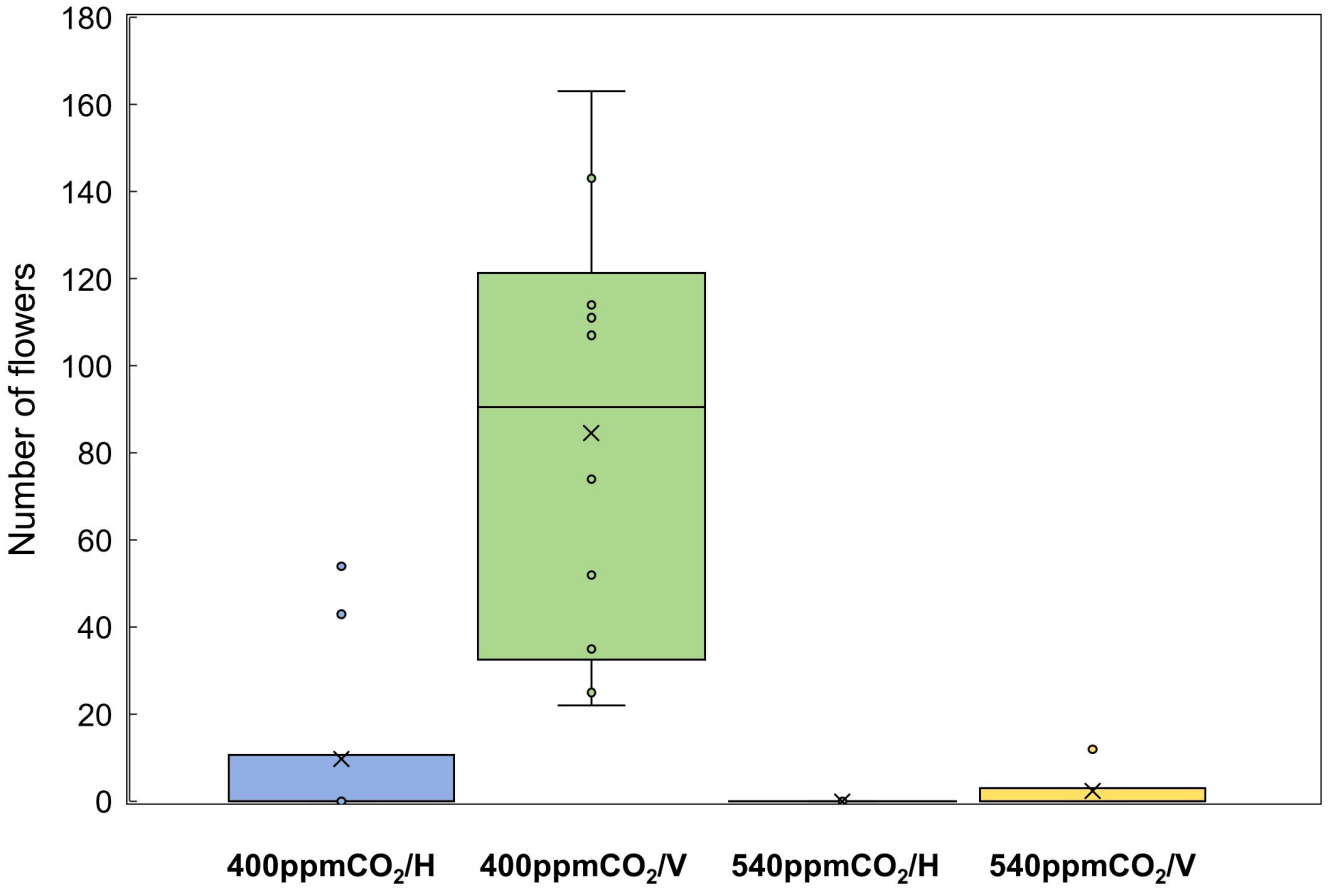
Number of flowers of *A. contorta* under two CO_2_ concentrations and two rhizome directions.

**Figure 9:**
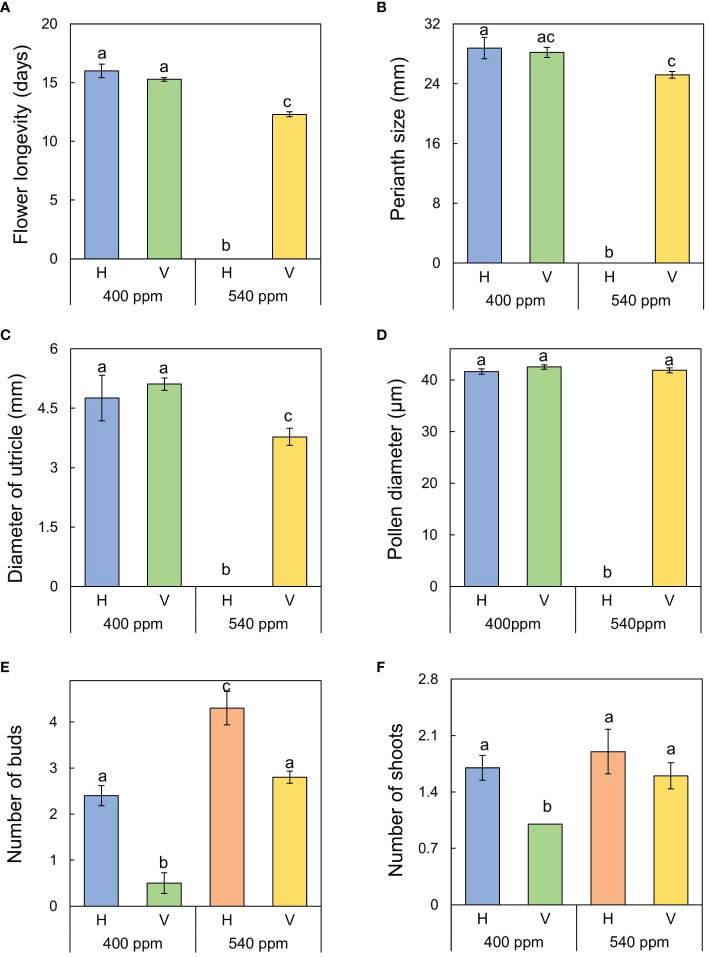
Reproductive (sexual and asexual) traits of *A*. *contorta* under two CO_2_ concentrations and two rhizome directions. **(A)** flower longevity, **(B)** perianth size, **(C)** diameter of utricle, **(D)** pollen diameter, **(E)** number of underground buds, **(F)** number of shoots. Letters on the graph indicate significant differences at the 5% level, based on Duncan’s test. Bars indicate standard errors.

Stems, internodes, and branches of *A. contorta* were significantly longer at 400ppmCO_2_ than at 540ppmCO_2_ (**Figure 6**). In addition, *A. contorta* produced a greater number of branches, and larger leaves at 400ppmCO_2_ than at 540ppmCO_2_. However, chlorophyll content was significantly higher at 540ppmCO_2_ than at 400ppmCO_2_. *A. contorta* with rhizome planted vertically showed longer stem, internode, and branches, thicker stem, higher number of branches and leaves, and larger leaves than individuals with rhizome planted horizontally (**Figure 6**). Stem length was longest in the 400ppmCO_2_/V (614.1 ± 67.5 cm), followed by 540ppmCO_2_/V (445.3 ± 40.5 cm), 400ppmCO_2_/H (326.8 ± 46.1 cm), and 540ppmCO_2_/H (216.2 ± 29.4 cm). Internode length was also longest in the 400ppmCO_2_/V (15.6 ± 1.2 cm), followed by 540ppmCO_2_/V (10.4 ± 0.8 cm), 400ppmCO_2_/H (10.4 ± 0.6 cm), and 540ppmCO_2_/H (7.5 ± 0.5 cm). Stem thickness was largest in the 400ppmCO_2_/V (2.83 ± 0.18 mm), followed by 540ppmCO_2_/V (2.64 ± 0.12 mm), 540ppmCO_2_/H (2.07 ± 0.23 mm), and 400ppmCO_2_/H (2.02 ± 0.16 mm). Rhizome direction influenced the number of branches and total branch length, with the largest values observed in the 400ppmCO_2_/V treatment (branches, 10.4 ± 0.9 cm; total branch length, 536.1 ± 39.2 cm), followed by the 540ppmCO_2_/V treatment (branches, 4.2 ± 0.4; total branch length, 220.8 ± 43.7 cm), the 400ppmCO_2_/H treatment (branches, 3.1 ± 0.5; total branch length, 135.7 ± 33.7 cm), and the 540ppmCO_2_/H treatment (branches, 2.8 ± 0.6; total branch length, 98.1 ± 19.1 cm). Number of leaves was largest in the 400ppmCO_2_/V (225.2 ± 22.4), followed by 540ppmCO_2_/V (170.1 ± 23.5), 400ppmCO_2_/H (102.1 ± 20.9), and 540ppmCO_2_/H (72.7 ± 9.7). Dried stem weight differed significantly under different CO_2_ concentrations (400ppmCO_2_, 5.35 ± 0.76 g; 540ppmCO_2_, 3.44 ± 0.45 g) and dried stem and leaf weights differed significantly under different rhizome directions (stem: H, 2.69 ± 0.33 g; V, 6.11 ± 0.68 g, leaves: H, 2.71 ± 0.76 g; V, 5.24 ± 0.53 g) (**Figures 7A–C**). The belowground/aboveground ratio was highest in the 540ppmCO_2_/H (0.61), followed by 400ppmCO_2_/H (0.39), 540ppmCO_2_/V (0.27), and 400ppmCO_2_/V (0.16) (**Figure 7D**). There was a 26-day difference in the FFD between conditions with 400ppmCO_2_ (occurring on June 13th) and conditions with 540ppmCO_2_ (occurring on July 9th). Under 400ppmCO_2_, flower longevity was extended, accompanied by an increased number of flowers (**Figure 8**) and larger flower size (perianth size and diameter of utricle) compared to 540ppmCO_2_ (**Figure 9**). Additionally, flowers exhibited earliest flowering, with a greater number of flowers, greater flower longevity, and larger perianth size (diameter of utricle) in vertical rhizome as opposed to horizontal rhizome. On the other hand, in terms of asexual reproductive traits, the number of underground buds and shoots was higher at 540ppmCO_2_ compared to 400ppmCO_2_.

All sexual reproductive traits were responsive to rhizome direction in both 400ppmCO_2_ and 540ppmCO_2_. Flower longevity was longest in 400ppmCO_2_/H (16.0 ± 0.6 days), followed by 400ppmCO_2_/V (15.3 ± 0.2 days), and 540ppmCO_2_/V (12.3 ± 0.2 days). Perianth size was largest in 400ppmCO_2_/H (28.8 ± 1.4 mm), followed by 400ppmCO_2_/V (28.2 ± 0.6 mm), and 540ppmCO_2_/V (25.2 ± 0.5 mm). Diameter of utricle was largest in 400ppmCO_2_/V (5.11 ± 0.08 mm), followed by 400ppmCO_2_/H (4.76 ± 0.12 mm), and 540ppmCO_2_/V (3.78 ± 0.18 mm).

**Figure 10:**
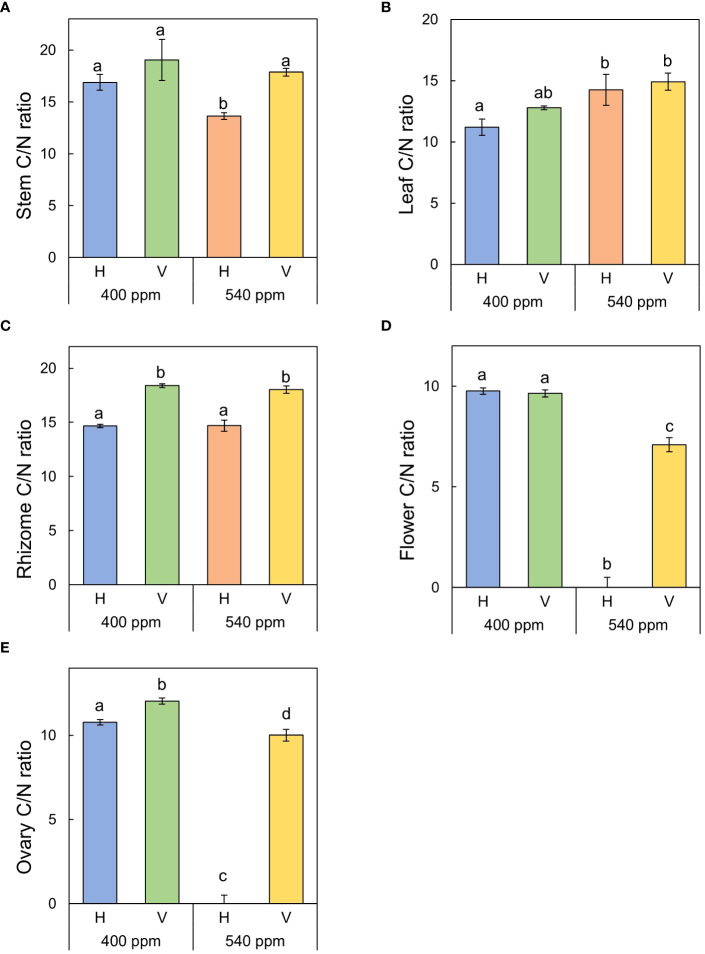
C/N ratio of each part of *A*. *contorta* under two CO_2_ concentrations and two rhizome directions. **(A)** stem C/N ratio, **(B)** leaf C/N ratio, **(C)** rhizome C/N ratio, **(D)** flower C/N ratio, **(E)** ovary C/N ratio.

Stem C/N ratio was highest in 400ppmCO_2_/V (19.06 ± 1.97), followed by 540ppmCO_2_/V (17.87 ± 0.37), 400ppmCO_2_/H (16.89 ± 0.76), and 540ppmCO_2_/H (12.3 ± 0.33) (**Figure 10**). The highest value of leaf C/N ratio was observed in 540ppmCO_2_/V (14.92 ± 0.69), followed by 540ppmCO_2_/H (14.24 ± 1.27), 400ppmCO_2_/V (12.78 ± 0.15), and 400ppmCO_2_/H (11.21 ± 0.66). The highest ratio of rhizome C/N ratio was found in 400ppmCO_2_/V (18.39 ± 0.17), followed by 540ppmCO_2_/V (18.04 ± 0.35), 540ppmCO_2_/H (14.70 ± 0.51), and 400ppmCO_2_/H (14.67 ± 0.16). Flower C/N ratio was highest in 400ppmCO_2_/H (9.76 ± 0.01), followed by 400ppmCO_2_/V (9.65 ± 0.14), and 540ppmCO_2_/V (7.09 ± 0.01). For ovary C/N ratio was highest in 400ppmCO_2_/V 12.04 ± 0.01), followed by 400ppmCO_2_/H (10.78 ± 0.01), and 540ppmCO_2_/V (10.02 ± 0.02).

The stigma reactions were the most intense at the 400ppmCO_2_/V and there were numerous bubbles at the 400ppmCO_2_/H (**Table 1**). The reactions of stigmas were not intense on at the 540ppmCO_2_/V with only a few bubbles on the stigma. Pollen viability rates were highest at 400ppmCO_2_/V (the viability range between 90.0 ± 1.2 - 97.0 ± 0.8%, reaching up to 100%), followed by 400ppmCO_2_/H (88.3 ± 1.7 - 93.3 ± 1.7%), and lowest at 540ppmCO_2_/V (71.7 ± 6.0 - 85.0 ± 2.9%, reaching up to 60%) in the observations conducted under an optical microscope (**Table 2**).

**Table 1:** Stigmatic receptivity of *A. contorta* under two CO_2_ concentrations and two rhizome directions.

	400ppmCO_2_	540ppmCO_2_
**Horizontal rhizome**	3.00 ± 0.00 (+++)	/
	3.00 ± 0.00 (+++)	/
	2.67 ± 0.33 (+++)	/
	2.33 ± 0.33 (++)	/
	2.33 ± 0.33 (++)	/
	2.00 ± 0.00 (++)	/
	2.33 ± 0.33 (++)	/
	2.67 ± 0.33 (+++)	/
	2.33 ± 0.33(++)	/
	2.67 ± 0.33 (+++)	/
**Vertical rhizome**	3.00 ± 0.00 (+++)	1.67 ± 0.33 (++)
	2.80 ± 0.13 (+++)	2.00 ± 0.00 (++)
	2.90 ± 0.10 (+++)	2.67 ± 0.33 (+++)
	3.00 ± 0.00 (+++)	2.00 ± 0.00 (++)
	3.00 ± 0.00 (+++)	1.33 ± 0.33 (+)
	2.80 ± 0.13 (+++)	1.00 ± 0.00 (+)
	2.90 ± 0.10 (+++)	1.33 ± 0.33 (+)
	2.50 ± 0.17 (++)	1.00 ± 0.00 (+)
	2.90 ± 0.10 (+++)	1.67 ± 0.33 (++)
	2.80 ± 0.13 (+++)	1.33 ± 0.33 (+)

+++, strong receptivity; ++, moderate receptivity; +, weak receptivity; /, no flower.

**Table 2:** Proportion of the stained pollen grains at *A. contorta* individuals (%).

	400ppmCO_2_	540ppmCO_2_
**Horizontal rhizome**	91.67 ± 1.67	/
	89.33 ± 0.67	/
	93.33 ± 3.33	/
	91.67 ± 1.67	/
	88.33 ± 1.67	/
	91.67 ± 1.67	/
	93.33 ± 1.67	/
	90.00 ± 2.89	/
	91.67 ± 1.67	/
	91.67 ± 1.67	/
**Vertical rhizome**	90.00 ± 1.18	81.67 ± 4.41
	97.00 ± 0.82	85.00 ± 2.89
	94.70 ± 1.58	71.67 ± 6.01
	93.50 ± 1.45	74.00 ± 2.08
	93.60 ± 1.48	80.00 ± 2.89
	96.00 ± 0.67	70.00 ± 2.89
	95.30 ± 1.52	75.00 ± 2.89
	95.50 ± 0.90	74.33 ± 2.33
	95.60 ± 1.54	85.00 ± 2.89
	94.50 ± 1.17	77.67 ± 1.45

n=30.

#### 3.2.3 Interactive effects of elevated CO_2_ concentrations and rhizome direction on growth and reproductive traits

Morphological and reproductive traits responded to differences in CO_2_ concentrations and rhizome directions (**Table 3**). For instance, number of shoots was only affected by CO_2_ concentrations, and stem thickness and dry leaf weight were only affected by rhizome directions. There were no interactive effects on dry rhizome weight. Morphological differences according to the CO_2_ concentrations were more apparent when the rhizome direction was different. We detected a significant interaction of CO_2_ concentrations and rhizome direction on number of branches, total branch length, single leaf area, total leaf area, dry stem weight, and sexual reproductive traits (number of flowers, flower longevity, perianth size, diameter of utricle, pollen grain size, stigmatic receptivity, and pollen viability).

**Table 3:** Two-way analysis of variance results for traits of *A. contorta* in the greenhouse experiments; *F* statistics are shown.

Traits of A. contorta		CO_2_ concentrations	Rhizome directions	CO_2_ concentrations × Rhizome directions
**Growth traits**	Stem length	8.483*	28.980***	0.368
	Internode length	24.155***	24.418***	2.001
	Stem thickness	0.181	15.232***	0.454
	Number of branches	17.564***	14.471***	31.465***
	Total branch length	15.189***	33.373***	9.405**
	Number of leaves	4.515*	30.746***	0.418
	Single leaf area	59.127***	20.065***	5.421*
	Total leaf area	22.367***	42.636***	1.509
	Chlorophyll content	5.491*	7.086*	0.038
	Dry stem weight	7.445*	28.980***	0.448
	Dry leaf weight	0.338	7.300*	1.354
	Dry rhizome weight	0.137	0.196	0.078
**Sexual reproductive traits**	Number of flowers	13.51**	17.581***	10.603**
	Flower longevity	37.136***	7.631**	10.089**
	Perianth size	22.862***	7.366*	8.214**
	Diameter of utricle	34.269***	9.295**	5.930*
	Pollen grain size	228.499***	233.732*	214.661***
	Stigmatic receptivity	180.523***	46.536***	20.401***
	Pollen viability.	1306.355***	724.843***	609.611***
**Asexual reproductive traits**	Number of buds	70.248***	46.035***	0.637
	Number of shoots	5.126*	3.821	1.263

The two treatments were CO_2_ concentrations (400ppmCO_2_ and 540ppmCO_2_) and rhizome directions (horizontal and vertical). *df*=1, 39 for traits. Significant effects are shown in boldface (*, *p* < 0.05; **, *p* < 0.01; ***, *p* < 0.001).

**Figure 11:**
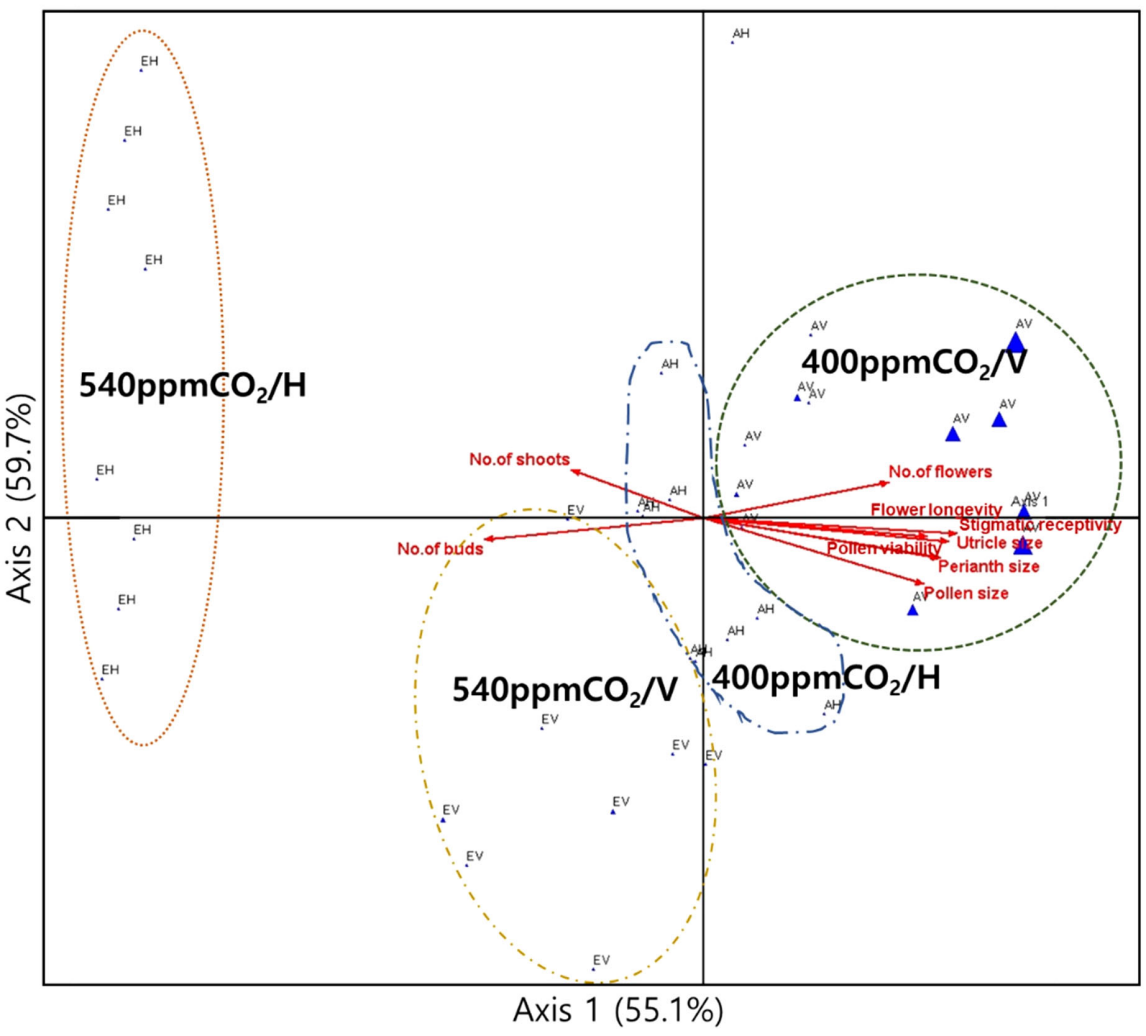
Canonical correlation analysis (CCA) plots to determine the relationships between growth and reproductive traits. The percentage (%) of each axis represents the explained variance. The dotted curves indicate the groups of individuals in the treatment groups, which are represented by the blue triangles. The arrows are strongly correlated with the axis.

Growth traits at 400ppmCO_2_ exhibited strong correlations with reproductive characteristics, including the number of flowers, flower longevity, perianth size, diameter of the utricle, pollen grain size, stigmatic receptivity, and pollen viability (**Figure 11**). Growth traits at 540ppmCO_2_ displayed strong correlations with the number of buds and shoots. Axes 1 and 2 accounted for 55.1% and 59.7% of the explained variance, respectively.

## 4 Discussion

### 4.1 Environmental factors and growth traits in *A. contorta*

We specifically focused on environmental factors and established a standard for assessing their significant influence on optimal flowering in mature *A. contorta*. During the reproductive phase, RLI was significantly lower (approximately 40%) than that during the vegetative phase, and the soil water content was notably higher than that during the vegetative phase (**Figures 1A, B**). Appropriate light intensity has been recognized as a significant driver of growth in mature *A. contorta*. Adequate light during the vegetative phase can enhance photosynthesis and establish a resource base for future reproductive efforts (Wimalasekera, 2019). However, excessive light such as RLI 100% can disrupt growing and flowering of mature *A. contorta* (Park et al., 2019; Wimalasekera, 2019). This inhibition can be attributed to various factors based on prior research, including photoinhibition, altered hormone regulation, and increased oxidative stress in plants exposed to excessively intense light conditions (Huang et al., 2019; Maai et al., 2020; Bassi and Dall'Osto, 2021; Roeber et al., 2021).

There was a negative correlation between RLI and soil water content (-0.512, *p* < 0.05) in both the vegetative and reproductive phase, with high light intensity identified as a factor contributing to a decrease in soil water content. Due to the higher RLI, *A. contorta* grown in arid soil conditions of the vegetative phase group exhibited inferior growth compared to the reproductive phase group and did not flower sufficiently. Adequate soil water is essential for nutrient uptake, photosynthesis, and overall plant health (Zainul et al., 2020; Chtouki et al., 2022). Without sufficient water, plants struggle to maintain their physiological processes and reach the reproductive phase, which is vital for successful flowering and reproductive success (Jomo et al., 2015; Chauhan et al., 2019).

The field survey also explored the role of soil nutrients, specifically aiming to identify the precise soil nutrient levels that are essential for optimal growth. While no significant differences in soil nutrient levels were observed between the vegetative and reproductive phase during the field survey (**Figure 1**), this suggests that the soil may contain an appropriate abundance of the necessary nutrients to support sufficient growth and flowering (Uchida, 2000; Pestana et al., 2005; Fathi, 2022). Prior research has consistently demonstrated that insufficient or excessive soil nutrient levels can significantly hinder growth and flowering processes (Uchida, 2000), highlighting the profound influence of nutrient availability on plant reproduction. While these vegetative and reproductive phases contain similar soil nutrient contents, the discrepancy in flowering can be attributed to differences in RLI and soil water content. These environmental factors were found to have a strong correlation with the growth characteristics and number of flowers of *A. contorta* (**Figure 4**). This implies that under suitable environmental conditions, *A. contorta* exhibits optimal growth, which is essential for flowering.

In addition to environmental factors from the field surveys, the results of the greenhouse experiment also demonstrated that elevated CO_2_ concentrations had a hindering effect on the growth of mature *A. contorta*. There was a significant decrease in stem length, internode length, leaf size, and branch development under 540ppmCO_2_ compared to 400ppmCO_2_ (**Figure 6**). Some plant responses, such as increased leaf thickness and mesophyll size, may enhance photosynthesis and growth (Prior et al., 2011; Zhang et al., 2013; Broughton et al., 2016; Choi et al., 2017; Chavan et al., 2019; Thruppoyil and Ksiksi, 2020). However, in other plants, specific changes in stomatal conductance and biochemical composition can counteract these positive effects, resulting in variable outcomes for growth and photosynthesis under elevated CO_2_ conditions (Poorter and Perez-Soba, 2002; Wang et al., 2012; Zheng et al., 2019). Elevated CO_2_ levels reduce stomatal conductance. This reduction decreases water loss but also limits CO_2_ diffusion into the leaf, affecting photosynthesis (Poorter and Perez-Soba, 2002; Wang et al., 2012). This reduction can affect the overall photosynthetic efficiency and, subsequently, growth. Interestingly, in our experiment at 540ppmCO_2_, even though there was a higher leaf chlorophyll content, the aboveground growth was not as favorable. This is because it is speculated that this additional resource allocation is directed towards other parts, such as rhizomes, root buds, and shoots. Such resource allocation can indicate trade-offs between biological growth and reproductive structures (Ziska et al., 2004; McPeek and Wang, 2007; Qin et al., 2022). Over time, plants exposed to elevated CO_2_ may undergo photosynthetic acclimation, where the initial increase in photosynthetic rate plateaus or decreases due to downregulation of Rubisco activity and other photosynthetic enzymes (Zhang et al., 2013; Broughton et al., 2016). This acclimation can limit growth benefits from increased CO_2_.

It exhibited higher C/N ratios in stem, rhizome, flower and ovary parts except for the leaves at the 400ppmCO_2_ condition (**Figure 10**). The different C/N ratios in leaves compared to other plant parts result from their influence on carbon and nitrogen levels. Carbon levels are regulated through leaves by affecting photosynthetic capabilities and carbohydrate export, while nitrogen quantities are modulated by their transport from stems to reproductive tissues (Yasumura, 2009). Plants possess the ability to independently govern carbon and nitrogen, a crucial trait in natural environments where external factors can readily disrupt the delicate carbon-nitrogen balance (Yasumura, 2009). In addition, elevated CO_2_ can affect nitrogen assimilation and utilization, leading to lower nitrogen content in tissues (Choi et al., 2017; Chavan et al., 2019). Since nitrogen is vital for chlorophyll and protein synthesis, its reduced availability can limit growth despite the increased photosynthetic potential.

Our study also revealed a noticeable difference in potassium absorption under elevated CO_2_ conditions (**Figure 5D**). Potassium serves as a catalyst for a range of enzymes and plays a crucial role in regulating the intracellular osmotic balance and facilitating the transport of membrane proteins (Ye et al., 2019). Additionally, it has a significant impact on carbohydrate transportation in plants, contributing to their overall metabolism and resilience to stress factors (Wang and Wu, 2013; Nieves-Cordones et al., 2019). At 540ppmCO_2_, the enhanced potassium absorption, likely attributable to its involvement in photosynthesis, may not have been the predominant factor affecting the flowering of *A. contorta*. These results underscore the intricate interplay between CO_2_ concentrations and various physiological and morphological traits in *A. contorta*.

### 4.2 Growth traits and their impact on reproductive traits in *A. contorta*

Our study revealed variations in the growth traits of *A. contorta*, such as thicker stems, longer internodes, increased leaf area, and more flowers in the reproductive phase (**Figure 2**). Before plants reach the flowering and reproduction stages, most plants go through a period of vegetative growth (Sola and Ehrlen, 2007). Vegetative and reproductive phases can be viewed as developmental phases where new organs continuously develop, each exhibiting distinct morphological traits such as internode length, leaf area, and cell size (Poethig, 2003; Huijser and Schmid, 2011; Wang et al., 2011). During this period, plants typically experience a rapid increase in their ability to perform photosynthesis and their overall size and mass (Sola and Ehrlen, 2007). Generally, plants develop reproductive organs only during the adult vegetative phase (Araki, 2001). This also aligns with the findings of Park et al. (2019), indicating that when an appropriate level of RLI is provided, *A. contorta* growth becomes more vigorous and has flowers. These observations underscore the importance of achieving a minimum level of growth during the transition from the vegetative to the reproductive phase, which is essential for successful flowering (Araki, 2001). Furthermore, the observed higher C/N ratio in the reproductive phase compared to the vegetative phase (**Figure 3**) carries substantial implications for nutrient availability and allocation (Uchida, 2000). As documented in earlier research (Zhang et al., 2022), plants may allocate resources toward nitrogen uptake when soil nitrogen levels exceed optimal ranges, potentially delaying or inhibiting the initiation of the flowering process. This phenomenon is often observed when plants adapt their resource allocation strategies to optimize their survival and reproduction under varying environmental conditions (Körner, 2015). Resource allocation in *A. contorta* refers to the distribution of limited resources among different physiological processes, including growth and reproduction (Hartmann et al., 2020). When an appropriate level of resources is available, as indicated by factors such as proper RLI and soil nutrient levels, plants allocate more resources to growth (Tandon et al., 2020). This allocation leads to thicker stems, longer internodes, and increased leaf areas, all of which are associated with a more vigorous growth phase (Tandon et al., 2020).

In greenhouse experiments, as in the field survey, plant growth in response to CO_2_ concentration contributed to the transition from the vegetative to the reproductive phase. Adequate vegetative growth, as observed at 400ppmCO_2_, characterized by long and thick stems and abundant leaves, was associated with a higher number of flowers (**Figures 6**, **10**). On the other side, at 540ppmCO_2_, insufficient growth was observed, which hindered flowering (**Figures 6**, **10**). In addition, at 400ppmCO_2_, flowering occurred earlier than under other conditions, while at 540ppmCO_2_, flowering was hindered. This may be associated with a greater allocation of resources to aboveground structures rather than belowground structures (**Figure 7D**). Elevated CO_2_ concentration also had an additional effect on plant flowering. These findings are in agreement with those of previous research (Jablonski et al., 2002), which also highlighted the influence of CO_2_ concentration on the reproductive traits of various plant species.

Our study also revealed significant reductions in flower longevity, decreased flower abundance, and smaller flower size under 540ppmCO_2_ (**Figure 9**). In the same context, several plant species (*Trifolium pratense, Capsicum annuum*, and *Cucurbita pepo*) exhibited a reduced number of flowers and shortened flower longevity under elevated CO_2_ conditions (Rusterholz and Erhardt, 1998; López-Cubillos and Hughes, 2016). Conversely, different plant species (*Phalaenopsis* Queen Beer, *Lotus corniculatus*, *Gerbera jamesonii*, and *Vitis vinifera* L.) produce a greater number of larger flowers and experience extended flower longevity in response to elevated CO_2_ conditions (Rusterholz and Erhardt, 1998; López-Cubillos and Hughes, 2016; Arrizabalaga-Arriazu et al., 2020). This highlights the divergent responses of plant species to elevated CO_2_, indicating that the impact varies depending on the specific plant type. Remarkably, traits like stigma receptivity and pollen viability also demonstrated increased vitality at the 400ppmCO_2_ condition, which may contribute to the reproductive success of *A. contorta*. This parallels a study on maize crop production, where elevated CO_2_ concentrations were found to have adverse effects not only on stigma receptivity and pollen viability but also on reproductive processes and yield (Prasad et al., 2006; Bokshi et al., 2021).

Moreover, it’s important to highlight that 540ppmCO_2_ appeared to be less favorable for overall growth, thereby promoting asexual reproduction which were the number of underground buds and shoots (**Figures 9E, F**). In the elevated CO_2_, the absence of flowering suggests that plants may have adopted an asexual reproduction strategy due to the challenging conditions for sexual reproduction. This trade-off highlights the flexibility of the reproductive strategies of mature *A. contorta*, suggesting dynamic resource allocation in response to different environmental cues to maximize overall reproductive fitness. This phenomenon aligns with observations in other plant species, such as *Cirsium arvense* and *Taraxacum officinale* (Ziska et al., 2004; McPeek and Wang, 2007). This shift towards increased asexual reproduction at 540ppmCO_2_ could potentially lead to reduced genetic diversity. Such a change in reproductive strategy has implications for genetic variation, since it may limit the introduction of new genetic variations typically associated with sexual reproduction (Nakonechnaya et al., 2012; Nam et al., 2020; Yu et al., 2021).

Therefore, based on vigorous growth, mature *A. contorta* (e.g. 400ppm/V) may strategically allocate resources to maximize its sexual reproductive success; otherwise, it (eg. 540ppm/H) may invest in asexual reproduction (**Figure 11**). In response to elevated CO_2_ concentrations, these findings suggest potential shifts in growth dynamics and reproductive patterns, while also shedding light on the broader ecological and evolutionary implications of resource allocation strategies in plant reproduction under the global environmental changes.

### 4.3 Interactive effects of elevated CO_2_ and rhizome direction on *A. contorta*

All morphological differences were influenced by either CO_2_ concentrations or the direction of the rhizomes (**Table 3**). A direct influence of CO_2_ concentration on the number of shoots was observed, indicating that the production of new shoots from the rhizome system was stimulated by elevated CO_2_ concentration. This finding aligns with previous research highlighting the impact of elevated CO_2_ on increased asexual reproduction (Ziska et al., 2004; McPeek and Wang, 2007).

Rhizome direction also has emerged as a key determinant influencing various aspects of plant morphology, particularly stem thickness and the weight of dry leaves (**Table 3**). This suggests that the rhizome direction plays a pivotal role in shaping the structural characteristics of the aboveground parts of the plant, potentially by affecting water, nutrient uptake and resource allocation (Silva et al., 2019). In situations where environmental conditions undergo rapid and unpredictable changes, the adaptability conferred by horizontal rhizomes becomes particularly advantageous (Berntson and Woodward, 1992). They are well-suited for scenarios where securing water resources swiftly is crucial, especially in arid regions. The distribution of rhizomes is recognized as critical for a plant’s ability to acquire essential water and nutrients (Miao et al., 1992).

In contrast, vertical rhizomes, which had more flowers than horizontal rhizome (**Figure 7**), can contribute to genetic stability and the promotion of evolutionary processes by facilitating the reliable transfer of genetic information from one generation to the next (Kobayashi, 2019). This is achieved through sexual reproduction, making them potentially more suited for stable and consistent environments (Liu et al., 2021). In the context of evolution, the adaptability of rhizome direction becomes a crucial factor (Berntson and Woodward, 1992). The documented enhancements in reproductive success associated with rhizome systems further support the idea that belowground factors play a significant role in influencing aboveground reproductive traits.

The influence of hormonal signaling pathways, such as auxin in the case of vertical rhizomes (Scott, 1972), in regulating growth responses to environmental cues further underscores the complex interplay between rhizome direction and environmental adaptability. Conversely, horizontal rhizomes may obstruct hormonal movement, leading to suboptimal growth conditions (Mellor et al., 2020). Therefore, under the same CO_2_ concentration, horizontal rhizomes had a more detrimental effect on growth and development than vertical rhizomes.

The most remarkable outcomes stemmed from the interaction between CO_2_ concentrations and rhizome directions (**Table 3**). This interaction led to substantial variations in a range of morphological and reproductive traits, such as branch number, total branch length, single leaf area, and total leaf area (**Figure 11**, **Table 3**). These changes signify an impact in the plant’s growth and structural characteristics when these factors are considered simultaneously. Furthermore, the interaction of CO_2_ concentrations and rhizome direction significantly influenced sexual reproductive traits (**Table 3**; the number of flowers, flower longevity, perianth size, diameter of utricle, pollen grain size, stigmatic receptivity, and pollen viability). These changes in reproductive traits have important implications for the plant’s overall reproductive success, suggesting that environmental variations can have far-reaching effects on its life cycle and ecological role. Therefore, our findings emphasize the intricate nature of plant responses to changes in CO_2_ concentrations and rhizome directions, providing valuable insights into the adaptability of *A. contorta*. Moreover, understanding the ecological implications of these responses is crucial, especially in the context of ongoing global environmental changes.

### 4.4 Limitations and challenges

Our research provides valuable insights into how environmental factors affect *A. contorta*’s growth and reproduction, yet it’s conducted in controlled settings that may not fully reflect natural ecosystem complexities. The study’s duration may also fall short of capturing *A. contorta*’s long-term adaptations to increased CO_2_, indicating the necessity for longer observation in future research. The variability in plant responses to elevated CO_2_ across different species, genotypes, and individuals highlights the challenge of generalizing findings and necessitates a broader spectrum of studies. Interactions with other environmental factors such as temperature, water availability, and nutrient levels further complicate the isolation of CO_2_ effects. Additionally, the detailed physiological and molecular mechanisms underlying CO_2_’s influence on plant growth remain partially understood, emphasizing the need for advanced, interdisciplinary approaches to unravel these complexities.

## 5 Conclusion

**Figure 12:**
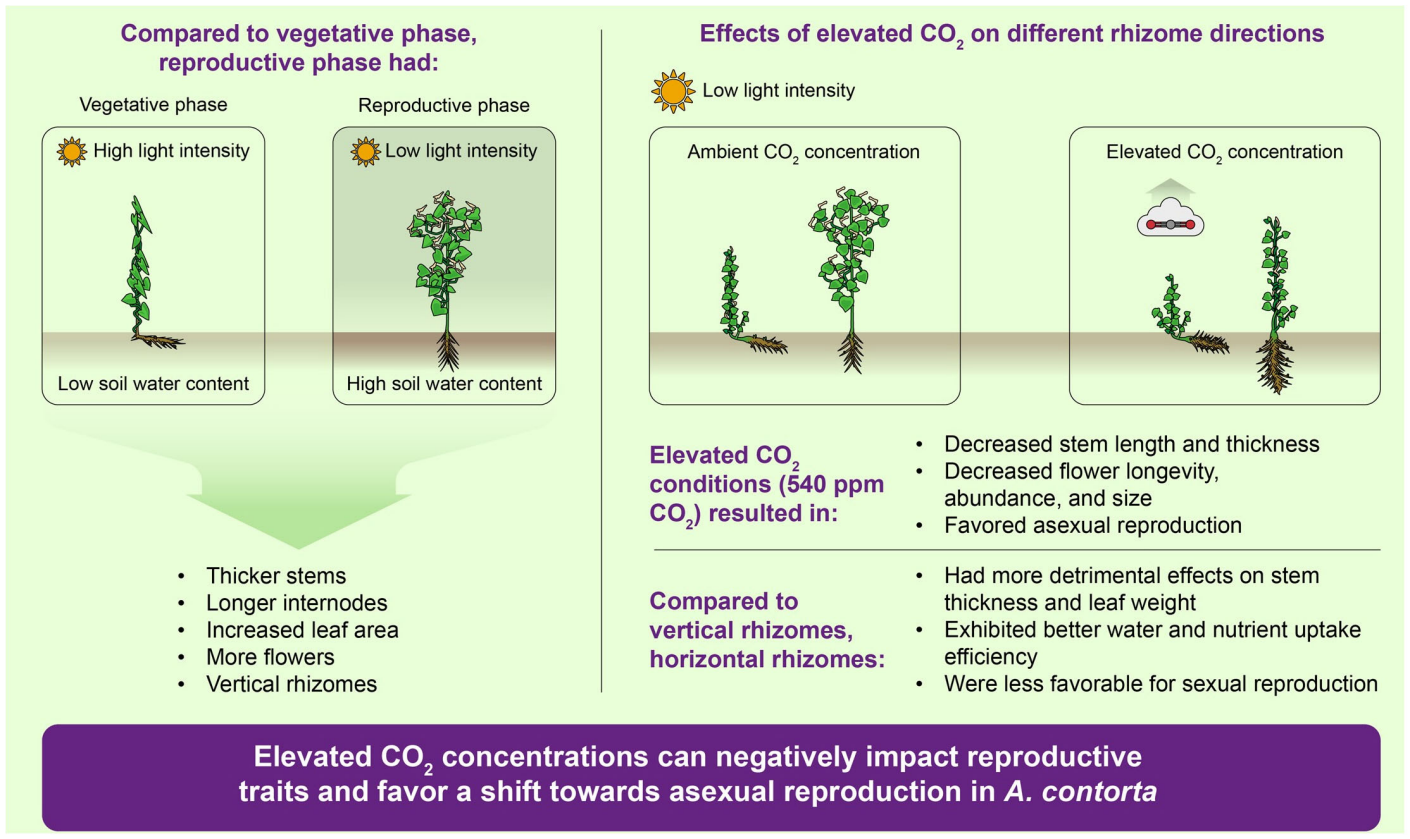
Comprehensive understanding of various factors that influence the asexual and sexual reproductions in mature *A. contorta*.

Our study provided a comprehensive understanding of the various factors that influence the probability of flowering in mature *A. contorta* (**Figure 12**). The research encompassed field surveys and greenhouse treatments, revealing the intricate interplay of environmental and physiological elements in shaping plant reproductive patterns. Field surveys have underscored the pivotal roles of light intensity, soil water content, and rhizome direction in influencing growth and flowering. Greenhouse experiments have revealed the interactive effects of CO_2_ concentration and rhizome conditions on flowering. CO_2_ concentration and rhizome direction also influenced growth traits, emphasizing the significance of substantial vegetative growth for successful flowering. Additionally, elevated CO_2_ concentrations exhibited diverse negative effects on the mature *A. contorta*’s reproductive traits, impacting flower size, longevity, stigma receptivity, and pollen viability, showing the complex interplay between environmental conditions and reproductive outcomes. Remarkably, our findings highlighted how environmental factors can inhibit growth and, in turn, hinder the sexual reproduction of mature *A. contorta*. Moreover, it triggers a shift towards increased asexual reproduction at elevated CO_2_ concentrations, potentially leading to reduced genetic diversity. These findings provide valuable insights into the adaptability and resource allocation strategies of mature *A. contorta* in response to ever-changing environmental cues. Moreover, our study sheds light on the broader ecological and evolutionary implications of these interactions, emphasizing the crucial role of environmental influences in shaping the reproductive patterns of mature *A. contorta*.

## Data availability statement

The original contributions presented in the study are included in the article/supplementary material. Further inquiries can be directed to the corresponding author.

## Author contributions

S-HP: Conceptualization, Data curation, Formal analysis, Investigation, Methodology, Visualization, Writing – original draft. JK: Conceptualization, Funding acquisition, Methodology, Project administration, Supervision, Writing – review & editing.
